# A Clinical Prediction Model for Genetic Risk in Children with GDD/ID: A Retrospective Study

**DOI:** 10.3390/pediatric18010001

**Published:** 2025-12-19

**Authors:** Yunshu Jiang, Ran Chen, Mengyin Chen, Luting Peng, Yuchen Zhao, Rong Li, Xiaonan Li

**Affiliations:** 1 Department of Child Healthcare, Children’s Hospital of Nanjing Medical University, Nanjing 210008, China; 18280215@stu.njmu.edu.cn (Y.J.); 13770604879@163.com (R.C.); lkjx0205@sina.com (M.C.); ltpeng@njmu.edu.cn (L.P.); 2 Department of Epidemiology, Harvard T.H. Chan School of Public Health, Boston, MA 02115, USA; yuchenzhao@hsph.harvard.edu

**Keywords:** global developmental delay, intellectual disability, gene, prediction model

## Abstract

Objectives: Global Developmental Delay (GDD) and Intellectual Disability (ID) are prevalent neurodevelopmental disorders with significant disability burden, and genetic factors play a crucial role in their etiology. This study aimed to develop and validate a clinical prediction model for identifying children with GDD/ID at high genetic risk, facilitating targeted genetic testing. Methods: We retrospectively analyzed clinical data of children with GDD/ID treated at Nanjing Children’s Hospital from January 2019 to December 2023. Children with comorbid Autism Spectrum Disorder (ASD) were excluded. The dataset was randomly split into training and validation sets (7:3 ratio). Lasso regression was used to identify potential predictive factors for positive genetic test results, followed by multivariable logistic regression to select independent predictors, which were incorporated into a nomogram. Model performance was evaluated by discrimination, calibration, and clinical utility using decision curve analysis in both sets. Results: Four independent predictors—craniofacial abnormalities, visceral abnormalities, physical growth abnormalities, and family history of ID—were identified. The resulting nomogram demonstrated an area under the curve (AUC) of 0.734., with good calibration and positive net benefit on decision curve analysis. Validation confirmed the reliability of the model. Conclusions: We developed a clinically applicable prediction model to identify high genetic risk among children with GDD/ID without ASD. This model may serve as a preliminary screening tool to assist clinicians in prioritizing genetic testing and improving diagnostic efficiency in clinical practice.

## 1. Introduction

Global developmental delay (GDD) and intellectual disability (ID) are neurodevelopmental disorders characterized by significant clinical and genetic heterogeneity [1]. GDD is diagnosed in children under the age of 5, while ID is diagnosed in children aged 5 years or older. GDD/ID is one of the most common pediatric neurological disorders, affecting approximately 1–3% of children worldwide [2]. In China, the annual incidence of GDD/ID is approximately 1.331‰, which translates to an estimated 136,000 new cases each year. GDD/ID poses severe threats to the physical and mental health of affected children, representing one of the leading causes of childhood disability and placing substantial psychological and economic burdens on families and society.

The etiology of GDD/ID is highly complex and can be broadly categorized into non-genetic and genetic factors. With improvements in living standards and healthcare measures, non-genetic factors such as infections, poisoning, trauma, and malnutrition have been significantly controlled. Consequently, the contribution of genetic factors has become increasingly prominent, with approximately 30–50% of GDD/ID cases attributable to genetic causes [3], a proportion that rises to two-thirds in cases of moderate to severe GDD/ID [4]. Genetic diagnosis of GDD/ID relies on genetic testing, with next-generation sequencing currently being the mainstream method due to its high diagnostic yield. However, the high cost and long turnaround time of such tests make them unsuitable for widespread screening.

Children with neurodevelopmental disorders who exhibit ≥1 special sign, particularly craniofacial anomalies and visceral malformations, have a significantly higher likelihood of pathogenic copy number variations (CNVs) [5], indicating that clinical markers are crucial for assessing genetic risk. Methods for the early identification of genetic diseases based on clinical features have been established for conditions such as Prader–Willi Syndrome (PWS), Silver–Russell Syndrome, and Williams Syndrome. However, these methods are limited to specific diseases and therefore have a narrow scope of application. Currently, effective approaches for broadly identifying the genetic risks of GDD and ID are still lacking. This study aims to develop a genetic risk prediction model that is applicable to all children with GDD/ID to help clinicians identify those at high genetic risk early. This would not only facilitate early diagnosis and precise treatment but also help prevent the recurrence of adverse reproductive events.

## 2. Materials and Methods

### 2.1. Study Design and Population

**Figure 1:**
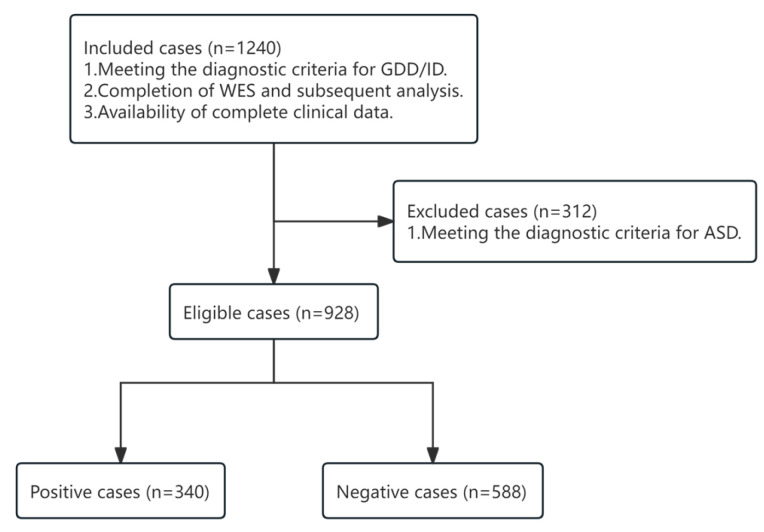
Flow Chart for Patient Selection.

A retrospective analysis was conducted on the clinical data of children with GDD/ID who visited the Department of Child Healthcare at Nanjing Children’s Hospital between January 2019 and December 2023. The inclusion criteria were as follows: (a) Meeting the diagnostic criteria for GDD/ID as outlined in the Diagnostic and Statistical Manual of Mental Disorders, Fifth Edition (DSM-5); (b) Having undergone whole exome sequencing (WES) with subsequent analysis; and (c) Availability of complete clinical data. Exclusion criteria were as follows: (a) Meeting the diagnostic criteria for Autism Spectrum Disorder (ASD) as defined by the DSM-5 (Figure 1).

The study planned to include 11 predictive factors, with each factor requiring data from 5–10 cases. Using the upper estimate of 10 cases per factor and a presumed positive rate of genetic test results at 30%, the minimum required sample size was calculated as 11 × 10 ÷ 30% = 367 cases.

Participation was voluntary, with informed consent obtained from their legal guardians. The study protocol received approval from the Medical Ethics Committee of Nanjing Medical University Affiliated Children’s Hospital (Approval No. 202110083).

### 2.2. Data Collection

#### 2.2.1. Diagnostic Criteria and Developmental Assessment

All diagnoses were confirmed by pediatricians specializing in child psychological and behavioral development at Nanjing Children’s Hospital. For children under the age of 5, the Gesell and Griffiths developmental scales were used to assess their Developmental Quotient (DQ). Global Developmental Delay was diagnosed if two or more developmental areas (including adaptability, gross motor skills, fine motor skills, language, and personal/social behavior) differed by more than two standard deviations. For children aged 5 years or older, the Chinese Wechsler Intelligence Scale was utilized to assess their Intellectual Quotient (IQ), while the Infants-Juvenile Social Life Ability Scale was used to evaluate social adaptive functioning. Children with an IQ below 70 and a social adaptive functioning score of 9 or lower were diagnosed with Intellectual Disability.

#### 2.2.2. Clinical Data Collection and Phenotyping

Clinical data were collected by clinicians and trained researchers at the Department of Child Healthcare (Table 1). Clinicians gathered information on the child’s gender, pregnancy history, birth history, and family history through medical history interviews. Craniofacial malformations and skin and hair abnormalities were assessed in reference to the Elements of Morphology (https://elementsofmorphology.nih.gov (accessed on 31 December 2023)). Physical examinations were conducted to preliminarily rule out visceral and skeletal malformations, with imaging studies performed for suspected cases to further confirm the diagnosis. Trained researchers conducted physical measurements of the children, including height and weight. Anthropometric measurements were used to diagnose physical growth abnormalities such as short stature, tall stature, low body weight, and obesity. All of these data were recorded in the Clinical Data Assignment Form (Table 2).

#### 2.2.3. Whole Exome Sequencing and Data Analysis

After obtaining informed consent, peripheral venous blood (2 mL each) was collected from the proband and their parents, and genomic DNA was extracted using standard methods. Exonic regions of all genes were captured using the MyGenostics GenCap Whole Exome Capture Kit (probe set P039-Exome; MyGenostics, Beijing, China), following the manufacturer’s instructions. Briefly, genomic DNA was randomly fragmented, ligated with Illumina PE adapters, and libraries were constructed using ligation-mediated Polymerase Chain Reaction (LM-PCR), followed by quality control. Subsequently, the libraries were hybridized with capture probes and sequenced in high throughput using the PE150 mode on the DNBSEQ-T7 platform (BGI, Shenzhen, China). Raw sequencing data underwent image and base recognition, with adapter sequences and low-quality reads removed. High-quality reads were aligned to the human reference genome (GRCh37/hg19) using BWA software (version 0.7.17), and single nucleotide variants (SNVs) and small insertions/deletions (Indels) were detected using GATK. All variants were functionally annotated using ANNOVAR software (version 2018-04-16). Sequencing quality control criteria included: mean sequencing depth > 100×, target region coverage (≥20×) ≥95%, and Q30 base proportion >85%; only samples meeting these criteria were included in downstream analysis.

#### 2.2.4. Variant Filtering and Pathogenicity Assessment

To identify rare variants, loci with minor allele frequency (MAF) ≥0.05 in East Asian populations in the 1000 Genomes Project, ExAC, and gnomAD databases were excluded. Pathogenicity of missense variants was predicted using SIFT, PolyPhen-2, and MutationTaster, with conservation scores provided by GERP++; potential effects of splice-site variants were assessed using SPIDEX (version 1.01.0). All candidate variants were queried in ClinVar (https://www.ncbi.nlm.nih.gov/clinvar/ (accessed on 31 December 2023)) and the Human Gene Mutation Database (HGMD, http://www.hgmd.cf.ac.uk/ (accessed on 31 December 2023)) to assess prior reports and pathogenicity. Subsequently, variants were classified according to the American College of Medical Genetics and Genomics (ACMG) guidelines by integrating population frequency [6], computational predictions, literature evidence, familial co-segregation, de novo occurrence, and gene-phenotype consistency. Specifically, de novo variants (validated via trio analysis) and familial co-segregation consistent with phenotype were considered PS2 and PP1 evidence, respectively; variants with functional experimental support were considered PS3, and those with negative functional results were considered BS3. All variants classified as pathogenic or likely pathogenic were validated by Sanger sequencing and co-segregation analysis in family samples to confirm inheritance patterns. Variants of uncertain significance (VUS) were reviewed by a second independent genetic analyst to minimize subjective bias. Final classifications were reviewed and confirmed by a multidisciplinary team comprising clinical geneticists, molecular geneticists, and pediatric clinicians. In this study, pathogenic and likely pathogenic variants were defined as “positive genetic findings,” whereas VUS were reported with recommendations for follow-up or further functional validation.

### 2.3. Statistical Analysis

#### 2.3.1. Data Processing and Descriptive Analysis

Data processing and statistical analysis were performed using R software (version 4.4.1, https://www.R-project.org/). Categorical variables were expressed as counts, and group comparisons were conducted using the χ2 test. When the expected frequency was less than 5, Fisher’s exact test was applied.

#### 2.3.2. Model Development and Validation

For model development, the glmnet package was used for fitting, with cross-validation performed via the cv.glmnet function to determine the optimal regularization parameter (λ.1se), thereby selecting relevant factors with nonzero regression coefficients. A logistic regression model was then constructed using the lrm function from the rms package, and a nomogram was generated to visually represent the model’s predictive outcomes.

#### 2.3.3. Performance Evaluation and Clinical Utility

The pROC package was employed to plot the receiver operating characteristic (ROC) curves and calculate the area under the curve (AUC) to evaluate the model’s discriminative ability. To further assess performance under class imbalance, precision–recall (PR) curves were generated, and the area under the PR curve was computed using the PRROC package. Calibration curves were generated using the calibrate function in the rms package to evaluate the agreement between predicted and observed outcomes, with calibration intercepts, slopes, and Brier scores reported. Decision curve analysis (DCA) was performed using the rmda package to quantify the clinical net benefit across a range of threshold probabilities. All statistical tests were two-sided, with a significance level set at *p* < 0.05.

## 3. Results

### 3.1. General Characteristics

A total of 928 children were included in the study. Based on genetic testing results, the children were divided into a positive group (340 cases) and a negative group (588 cases). There were no statistically significant differences between the two groups regarding offspring of advanced maternal age (AMA), assisted reproductive technology (ART) offspring, and premature infant (all *p* ≥ 0.05). However, there were statistically significant differences between the two groups in terms of gender, craniofacial malformations, skeletal abnormalities, skin and hair abnormalities, visceral abnormalities, epilepsy, physical development abnormalities and family history of ID (all *p* < 0.05) (Table 3). All the data were randomly divided into a training set (649 cases) and a validation set (279 cases) at a ratio of 7:3. The clinical characteristics between the two groups were compared using the chi-square test, and the results showed no statistically significant differences in any variables (all *p* ≥ 0.05) (Table 4), indicating good consistency in clinical characteristics between the two groups.

### 3.2. Screening for Predictive Factors

**Figure 2:**
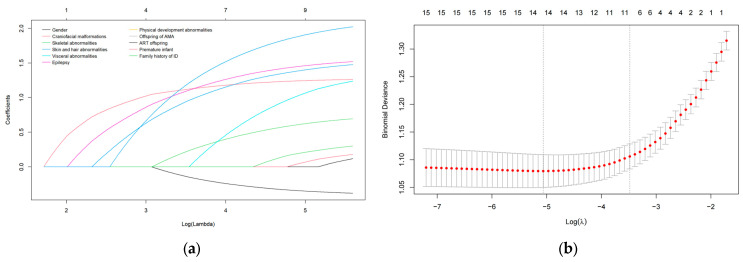
LASSO Regression for Variable Selection. (**a**) Coefficient profiles of predictors. (**b**) Cross-validation for tuning parameter (λ). In (**a**), the colored lines show the paths of the coefficients of each predictor in LASSO regression, shrinking gradually to zero as the regularization parameter (Log Lambda) increases. In (**b**), the red dots mark the optimal Lambda value determined by cross-validation, where the model achieves the best balance between deviance and complexity. The gray line segments indicate the standard error of deviance at each point.

Lasso regression analysis selected six non-zero coefficient predictors (Figure 2): gender, craniofacial malformations, skin and hair abnormalities, visceral abnormalities, physical development abnormalities, and family history of ID. A multivariate logistic regression analysis showed that craniofacial malformations, visceral abnormalities, physical development abnormalities, and family history of ID were statistically significant (all *p* < 0.05) (Table 5).

### 3.3. Risk Prediction Nomogram Development

**Figure 3:**
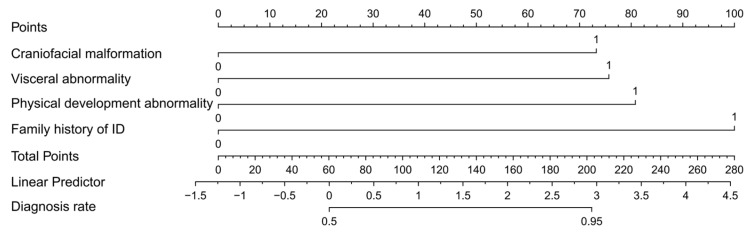
Nomogram for Genetic Risk in Children with GDD/ID.

Based on the four independent predictors mentioned above, a clinical prediction model for the genetic risk of GDD/ID in children was established and a nomogram was created (Figure 3). The formula for the logistic regression model is Equation (1):

Logit(P) = −1.24 + 1.51 × craniofacial malformation + 1.56 × visceral abnormality + 1.67 × physical development  abnormality + 2.07 × family history of ID(1)

### 3.4. Predictive Accuracy and Net Benefit of the Nomogram

**Figure 4:**
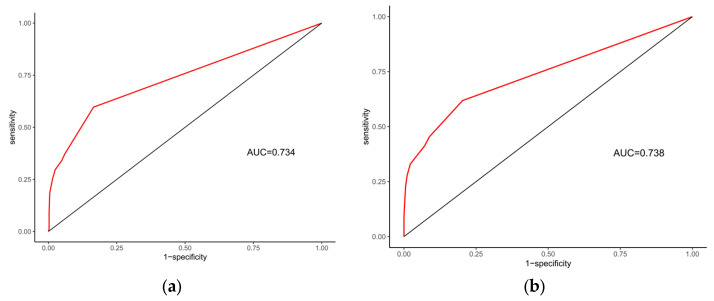
ROC Curves of the Model. (**a**) Training set. (**b**) Validation set. The red line is the ROC curve of the model, showing the relationship between sensitivity and 1–specificity. The black diagonal line is the reference line, representing the performance of a random classifier (AUC = 0.5).

**Figure 5:**
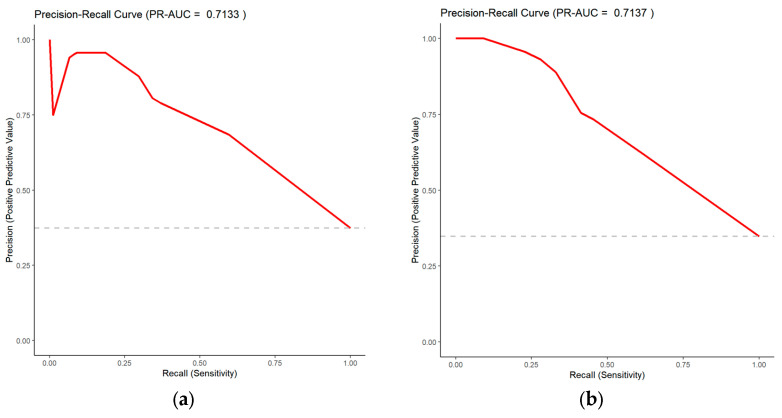
Precision–Recall Curves of the Model. (**a**) Training set. (**b**) Validation set. The red line is the Precision–Recall (PR) curve of the model, showing the relationship between precision and recall across different classification thresholds. The grey dashed line is the reference line, representing the prevalence of the positive class in the dataset.

**Figure 6:**
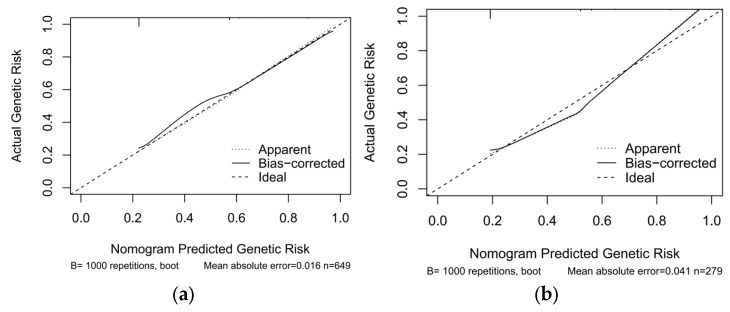
Calibration Curve for Predicting Probability. (**a**) Training set. (**b**) Validation set.

**Figure 7:**
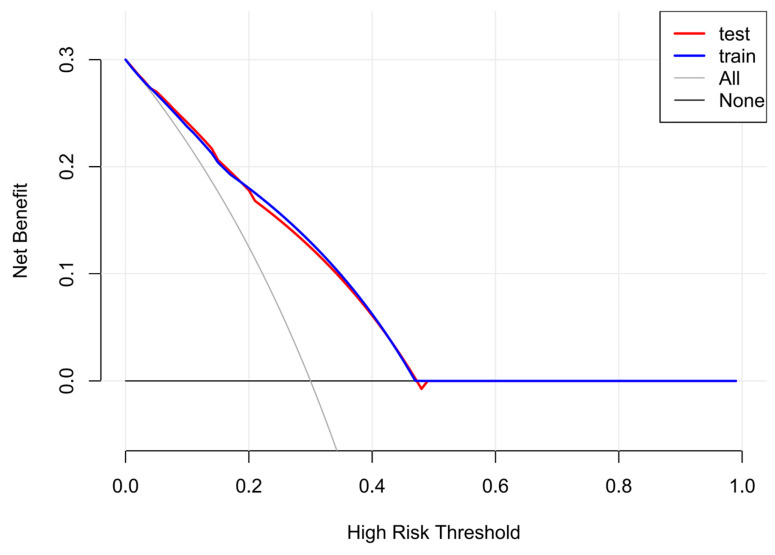
Decision Curve Analysis of the Model.

The predictive performance and clinical applicability of the nomogram were comprehensively evaluated in both the training and validation sets. The model demonstrated robust discriminative ability and moderate predictive accuracy across both datasets (Figure 4, Table 6). Specifically, the model achieved an AUC of 0.734 (95% CI: 0.698–0.771) in the training set, with high specificity (0.835) and moderate sensitivity (0.597). In the validation set, the AUC was 0.738 (95% CI: 0.679–0.796), showing similar sensitivity (0.619) and good specificity (0.797). The overall accuracies for the training and validation sets were 0.745 and 0.735, respectively, indicating strong model stability without evident overfitting. The PR-AUC was 0.7133 for the training set and 0.7137 for the validation set (Figure 5), further confirming the model’s stable predictive ability in distinguishing genetically positive and negative cases. In terms of calibration performance, the model demonstrated excellent agreement in both datasets (Figure 6, Table 7). The Brier scores were low (training: 0.180; validation: 0.175). The calibration intercept and slope were very close to the ideal values (training: 1.64 × 10^−14^, 1.000; validation: −0.199, 0.994). The calibration curves closely followed the ideal diagonal line, indicating high consistency between predicted probabilities and observed outcomes. Furthermore, DCA showed that the model achieved positive net benefits across a wide range of threshold probabilities, strongly supporting its clinical utility in predicting genetic risk among children with GDD/ID (Figure 7).

## 4. Discussion

### 4.1. Genetic Heterogeneity of GDD/ID and the Necessity of Risk Assessment

GDD/ID exhibits an extremely complex genetic basis, with etiologies involving multiple systems and numerous gene variants. For example, the “Deciphering Developmental Disorders (DDD)” study has identified 2940 genes definitively associated with developmental disorders, fully illustrating the remarkable genetic heterogeneity of this condition. This complexity makes it difficult to establish a simple correspondence between clinical phenotypes and genotypes: identical phenotypes may arise from different genetic mutations, while mutations in the same gene (pleiotropy) can also lead to diverse phenotypic manifestations. In this study, more than half of the patients with positive genetic findings presented multisystem abnormalities beyond the nervous system, and this high frequency of pleiotropic features provides a solid theoretical basis for integrating multiple clinical characteristics into a systematic genetic risk assessment. Against this background, the early and accurate identification of GDD/ID patients at high genetic risk is of particular importance. Based on four key clinical features, this study successfully constructed and validated a genetic risk prediction model for GDD/ID patients without ASD. The model demonstrated moderate but stable predictive performance and good calibration, supporting its potential value as a preliminary tool for clinical stratification.

### 4.2. Biological Basis of Model Predictors

Craniofacial malformations occurred in 24.5% of cases in this study cohort, representing the most common associated anomaly, with a significantly higher incidence in the genetically positive group (*p* < 0.001), consistent with recent multicenter studies in Chinese children [7]. On the embryonic developmental timeline, the central nervous system (CNS) and craniofacial structures share the same developmental window, both beginning at the fifth week of embryogenesis [8]. Anatomically, the cranial base provides both a structural platform for brain development and a connection for craniofacial morphogenesis [9]. At the molecular level, signaling pathways regulate the development of both the CNS and craniofacial structures. Wnt, Sonic Hedgehog (SHH), fibroblast growth factor (FGF), and bone morphogenetic protein (BMP) are classical regulatory molecules involved in craniofacial skeletal and dental development [10,11,12,13], while they also play crucial roles in establishing neural tube polarity, regulating neural progenitor cell proliferation and differentiation, and promoting dendritic development [14,15,16,17]. Abnormalities in any of these signaling pathways may disrupt the development of both the craniofacial structures and the CNS.

Visceral abnormalities, encompassing the cardiovascular, genitourinary, and digestive systems, are also key predictive indicators. Congenital heart disease (CHD) is the most commonly observed visceral abnormality. Although survival rates in children with CHD have improved, the incidence of neurodevelopmental disorders (NDD) remains considerable (approximately 20%) [18]. Among patients with CHD and comorbid NDD, 10% were found to carry deleterious de novo mutations, many of which are highly expressed in the heart and brain and enriched in pathways such as chromatin modification, transcriptional regulation, and Notch and Wnt signaling [19]. CNVs account for about 10% of cases in children with CHD, and the CNV detection rate increases in CHD patients with NDD, suggesting that gene dosage imbalances may be a potential cause of adverse neurocognitive outcomes [20].

This study indicates a significant association between physical growth abnormalities and genetic etiology (OR = 5.31). This association may arise from multiple genetic mechanisms: in chromosomal disorders, exemplified by trisomy 21, the dosage effects of genes such as DYRK1A can directly affect skeletal development, while concomitant structural or functional cardiac abnormalities may further limit the child’s growth potential [21,22]. In the context of monogenic disorders, represented by PWS, the physical phenotype exhibits an age-related dynamic evolution: during infancy, feeding difficulties and growth retardation predominate; in childhood, hyperphagia and central obesity gradually emerge; during adolescence, the degree of obesity further increases, often accompanied by short stature and hypogonadism, ultimately resulting in the characteristic short-statured obese phenotype in adulthood [23,24].

A positive family history of intellectual disability (OR = 7.90) was identified as the most significant predictor. This finding is consistent with results from large-scale family studies [25], highlighting the pivotal role of genetic burden. This predictor has been incorporated as a key factor in both the de Vries scoring system and the PredWES model, further underscoring its reliability [26,27].

### 4.3. Potential Predictive Factors Not Included

In addition to the four core predictors ultimately included in the model, this study also identified potential associations between genetic risk for GDD/ID and factors such as preterm birth, ART, and AMA. These factors may be related to complex interactions between genetic and environmental influences, such as epigenetic alterations following ART or an increased risk of chromosomal nondisjunction associated with AMA [28,29,30]. Although these variables were not included in the final model due to sample size limitations, their predictive value warrants further investigation in larger future cohorts.

### 4.4. Comparative Advantages over Existing Prediction Tools

Compared with existing predictive tools, the nomogram model developed in this study demonstrates clear advantages in methodology, predictive performance, and clinical applicability. The de Vries score is primarily designed for assessing submicroscopic telomeric rearrangements, with a relatively limited scope of application [26]. When its cutoff value is set at ≥6 points, although specificity is high (0.88), sensitivity decreases markedly, resulting in a missed diagnosis rate of up to 44%. In contrast, the continuous risk probabilities produced by our model are applicable to a broader range of genetic etiologies in GDD/ID, offering a more balanced overall discriminative performance. Compared with the machine-learning–based PredWES model, which incorporates hundreds of Human Phenotype Ontology (HPO) terms and employs a complex Bayesian logistic regression algorithm, the latter achieved an AUC of 0.76 and a Brier score of 0.175 [27]. Our model achieves comparable discriminative performance but offers advantages in simplicity and interpretability: it includes only four highly integrative and easily interpretable clinical features, forming a transparent and intuitive logistic regression framework that clearly presents each variable’s β coefficient and odds ratio. This approach avoids the common “black box” issue of machine learning models, making it more likely to gain clinicians’ trust. Furthermore, the final presentation of the model as a nomogram relies solely on information obtained from routine physical examinations and medical history, without the need for complex computational platforms or advanced application interfaces. This design significantly enhances its accessibility in clinical practice, particularly facilitating its implementation in primary healthcare settings with limited medical resources.

### 4.5. Theoretical Basis and Clinical Significance of Excluding ASD Comorbid Patients

The comorbidity of GDD/ID and ASD is not uncommon, with previous studies reporting a prevalence ranging from 4.2% to 32.9% [31,32,33,34]. However, children with isolated GDD/ID differ markedly from those with comorbid ASD in terms of neurodevelopmental mechanisms, core phenotypic characteristics, and potential genetic etiologies. Our univariate analysis revealed that, compared with individuals with isolated GDD/ID, children with comorbid ASD showed significant differences in key clinical features such as gender, craniofacial malformations, skin and hair abnormalities, visceral abnormalities, and physical development abnormalities (all *p* < 0.05) (Table S1). These phenotypic differences provide statistical justification for considering the two as heterogeneous subgroups. To quantify the impact of this heterogeneity on model performance, we performed a supplementary analysis. This involved expanding the cohort to include patients with comorbid ASD and re-applying the full model-development pipeline. Within this combined patient cohort, LASSO regression was utilized to perform penalized variable selection (Figure S1). Multivariate logistic regression then served to identify the cohort’s independent predictors (Table S2). Based on these factors, the supplementary predictive model was constructed and is visually presented as a nomogram in Figure S2.The performance of the final supplementary model was subject to comprehensive evaluation. Its discriminative ability was assessed via ROC curve (Figure S3) and PR curve (Figure S4). Classification outcomes and detailed performance metrics were presented in the confusion matrix and summarized in Tables S3 and S4. Finally, the model’s calibration was evaluated using the calibration curve (Figure S5), with its specific calibration parameters documented in Table S5. The results showed a marked decline in model discrimination, with the AUC dropping from 0.73–0.74 in the original model to 0.6745 (95% CI: 0.648–0.701) (Table S4), the PR-AUC decreasing from 0.71 to 0.667, accompanied by decreases in sensitivity, specificity, and goodness-of-fit, while the clinical net benefit also deteriorated (Figure S6). Therefore, when applying this model in clinical settings, it is essential to first exclude ASD comorbidity through standardized assessment scales such as the Aberrant Behavior Checklist (ABC), Childhood Autism Rating Scale (CARS), and Autism Diagnostic Observation Schedule (ADOS). For younger children with atypical symptoms, continuous monitoring of their sensorimotor, language–cognitive, and social–interaction developmental trajectories is recommended.

### 4.6. Clinical Implementation Pathway and Recommendations

Based on the findings of this study, we propose the following clinical decision pathway for children with GDD/ID: first, clinicians should rigorously assess and exclude comorbid ASD. For children without ASD, further evaluation of the four key clinical features should be performed, and individualized genetic risk probabilities should be calculated using the nomogram. For children with a predicted probability ≥0.40, it is recommended to follow ACMG guidelines and use trio whole-exome sequencing or whole-genome sequencing as the first-line diagnostic strategy. For children with a predicted probability of <0.40 who are not classified as high risk, a stepwise diagnostic approach can be considered, starting with chromosomal microarray analysis and gradually advancing genetic testing, while closely monitoring the evolution of key clinical features during follow-up.

## 5. Conclusions

Given the central role of genetic factors in the etiology of GDD/ID, early identification of individuals at high genetic risk has become a key step toward achieving precise etiological diagnosis. This study focused on children with GDD/ID without comorbid ASD and successfully developed and validated a genetic risk prediction model based on four key clinical features: craniofacial abnormalities, visceral abnormalities, physical growth abnormalities, and a family history of ID. The model demonstrated moderate yet robust discriminative performance and can serve as a practical preliminary screening tool to help clinicians effectively identify children at genetic risk and inform subsequent genetic testing strategies. This tool is expected to play an important role in primary healthcare settings or contexts with limited clinical experience, assisting in improving the efficiency of etiological diagnosis and the rational use of resources for GDD/ID.

## 6. Limitations and Perspectives

This study has several limitations: first, as a single-center retrospective study, it may be subject to selection and information biases and is constrained by the completeness of clinical records. Second, the study cohort was primarily drawn from the Chinese population, and its generalizability warrants further validation in populations of different ethnicities and regions. The exclusion of patients with comorbid ASD to control for phenotypic heterogeneity also limits the applicability of the model to broader neurodevelopmental disorder populations. Finally, genetic diagnosis relied mainly on WES, which may miss some structural variants or pathogenic mutations in non-coding regions, which could potentially lead to an underestimation of the detection rate of genetic etiologies. Future studies should focus on testing multicenter prospective cohorts for external validation, integrate multiple techniques such as genome sequencing and chromosomal microarray analysis to enhance the genetic assessment system, and explore the development of a comprehensive risk prediction model covering both isolated GDD/ID and cases with comorbid ASD, thereby improving the clinical utility and generalizability of the tool.

## Figures and Tables

**Table 1:** Clinical Data Description Table.

Clinical Characteristics	Description
Gender	female, male
Abnormal head circumference	macrocephaly, microcephaly
Eyebrow abnormalities	arched eyebrows, unibrow, thick eyebrows, sparse eyebrows, et al.
Eye abnormalities	hypertelorism, epicanthus, strabismus, ptosis, exophthalmos, enophthalmos, heterochromatic sclera, et al.
Ear abnormalities	prominent ears, low-set ears, posteriorly rotated ears, large ears, auricular deformities, pointed ears, accessory auricles, et al.
Nasal abnormalities	low nasal bridge, high nasal bridge, upturned nostrils, wide nasal root, et al.
Lip and palate abnormalities	cleft palate, high arched palate, thin upper lip, thick upper lip, cleft lip, long philtrum, downturned mouth corners, et al.
Dental abnormalities	malocclusion, geminated teeth, tooth agenesis, et al.
Mandibular abnormalities	micrognathia, et al.
Trunk skeletal abnormalities	pectus carinatum, pectus excavatum, shield chest, scoliosis, rickets, et al.
Limb skeletal abnormalities	polydactyly, brachydactyly, clinodactyly, limb asymmetry, et al.
Skin and hair abnormalities	simian crease, Mongolian spots, café-au-lait spots, hypertrichosis, alopecia, abnormal hair color, et al.
Visceral abnormalities	cardiovascular, urinary, reproductive, gastrointestinal abnormalities, et al.
Epilepsy	-
Physical development abnormalities	short stature, tall stature, low body weight, obesity
Offspring of AMA	born to a mother who is typically aged 35 years or older at the time of childbirth
ART offspring	born through assisted reproductive technology
Premature infant	born before 37 weeks of gestation
Family history of ID	in immediate family members (such as parents and siblings)

Note: ID = intellectual disability; AMA = advanced maternal age; ART = Assisted Reproductive Technology.

**Table 2:** Clinical Data Collection Form.

Variables	Values	
Gender	Female	Male
Craniofacial malformations	None	Yes
Skeletal abnormalities	None	Yes
Skin and hair abnormalities	None	Yes
Visceral abnormalities	None	Yes
Epilepsy	No	Yes
Physical development abnormalities	No	Yes
Offspring of AMA	No	Yes
ART offspring	No	Yes
Premature infant	No	Yes
Family history of ID	No	Yes

Note: Any abnormality in one of the following is considered a craniofacial malformation: head circumference, eyebrows, eyes, ears, nose, lips and palate, teeth, or jaw. Any abnormality in one of the following is considered a skeletal abnormality: limbs or trunk bones. ID = intellectual disability; AMA = advanced maternal age; ART = Assisted Reproductive Technology.

**Table 3:** General Characteristics of the Patients in Negative and Positive Groups.

Variables		Negative Group (*n* = 588)	Positive Group (*n* = 340)	*χ*2-Value	*p*-Value
Gender	Male	445	208	21.733	<0.001
	Female	143	132		
Craniofacial malformations	+	72	155	129.622	<0.001
	-	516	185		
Skeletal abnormalities	+	9	18	10.802	0.001
	-	579	322		
Skin and hair abnormalities	+	11	41	42.275	<0.001
	-	577	299		
Visceral abnormalities	+	12	53	60.701	<0.001
	-	576	287		
Epilepsy	+	2	7	6.626	0.014
	-	586	333		
Physical development abnormalities	+	22	80	86.223	<0.001
	-	566	260		
Offspring of AMA	+	3	3	0.464	0.674
	-	585	337		
ART offspring	+	4	5	1.401	0.300
	-	584	355		
Premature infant	+	6	9	3.585	0.058
	-	582	331		
Family history of ID	+	7	28	29.459	<0.001
	-	581	258		

Note: + = yes; - = no; ID = intellectual disability; AMA = advanced maternal age; ART = Assisted Reproductive Technology.

**Table 4:** General Characteristics of All Patients in the Training Set and Validation Set.

Variables		Training Set (*n* = 649)	Validation Set (*n* = 279)	*χ*2-Value	*p*-Value
Genetic test result	Positive	243	97	0.602	0.438
	Negative	406	182		
Gender	Male	459	194	0.133	0.716
	Female	190	85		
Craniofacial malformations	+	163	64	0.500	0.479
	-	486	215		
Skeletal abnormalities	+	15	12	2.735	0.098
	-	634	267		
Skin and hair abnormalities	+	38	14	0.259	0.611
	-	611	265		
Visceral abnormalities	+	46	19	0.023	0.879
	-	603	260		
Epilepsy	+	7	2	0.266	0.732
	-	642	277		
Physical development abnormalities	+	64	38	2.818	0.093
	-	585	241		
Offspring of AMA	+	5	1	0.516	0.675
	-	644	278		
ART offspring	+	6	3	0.046	1.000
	-	643	276		
Premature infant	+	12	3	0.735	0.572
	-	637	276		
Family history of ID	+	23	12	0.308	0.579
	-	626	267		

Note: + = yes; - = no; ID = intellectual disability; AMA = advanced maternal age; ART = Assisted Reproductive Technology.

**Table 5:** Multivariate Logistic Regression Analyses for Screening Predictors.

Variables	*β* (SE)	OR (95% CI)	*p*-Value
Craniofacial abnormalities	1.51 (0.21)	4.55 (2.78–7.44)	<0.0001
Visceral abnormalities	1.56 (0.44)	4.79 (2.02–11.38)	0.0003
Physical development abnormalities	1.67 (0.36)	5.31 (2.69–10.47)	<0.0001
Family history of ID	2.07 (0.54)	7.90 (2.79–22.40)	0.0001

Note: ID = intellectual disability.

**Table 6:** Performance Metrics of the Model.

Metric	Training Set	Validation Set
True Negative	339	145
False Negative	98	37
False Positive	67	37
True Positive	145	60
Accuracy	0.745	0.735
Sensitivity	0.597	0.619
Specificity	0.835	0.797
Positive Predictive Value	0.684	0.619
Negative Predictive Value	0.776	0.797
AUC (95% CI)	0.734 (0.698–0.771)	0.738 (0.679–0.796)
PR-AUC	0.7133	0.7137
Threshold	0.395	—

Note: AUC = Area Under the Receiver Operating Characteristic Curve; PR-AUC = Area Under the Precision–Recall Curve.

**Table 7:** Calibration and Predictive Performance of the Model.

Metric	Training Set	Validation Set
Brier	0.180	0.175
Calibration Intercept	1.64 × 10^−14^	−0.199
Calibration Slope	1.000	0.994
95% CI of Slope	0.817–1.196	0.726–1.288

## Data Availability

Data supporting reported results can be found at corresponding authors.

## Supplementary Materials

The following supporting information can be downloaded at: https://www.mdpi.com/article/10.3390/pediatric18010001/s1, Table S1: Univariate analysis comparing ASD + GDD/ID and ID-only groups. Figure S1: LASSO Regression for Variable Selection. Table S2: Multivariate Logistic Regression Analyses for Screening Predictors. Figure S2: Nomogram for Genetic Risk in Children with GDD/ID. Figure S3: ROC Curves of the Model. Figure S4: Precision-Recall Curves of the Model. Table S3: Confusion Matrix of the Model. Table S4: Performance Metrics of the Model. Figure S5: Calibration Curve for Predicting Probability. Table S5: Calibration and Predictive Performance of the Model. Figure S6: Decision Curve Analysis of the Model.

## Abbreviations

The following abbreviations are used in this manuscript:

GDD	Global developmental delay
ID	Intellectual disability
ASD	Autism spectrum disorder
AUC	Area under the curve
CNVs	Copy number variations
PWS	Prader–Willi syndrome
DSM-5	Diagnostic and Statistical Manual of Mental Disorders, Fifth Edition
WES	Whole Exome Sequencing
DQ	Developmental quotient
IQ	Intellectual quotient
AMA	Advanced maternal age
ART	Assisted reproductive technology
LM-PCR	Ligation-mediated Polymerase Chain Reaction
SNVs	Single nucleotide variants
Indels	Insertions/deletions
MAF	Minor Allele Frequency
HGMD	Human Gene Mutation Database
ACMG	American College of Medical Genetics and Genomics
VUS	Variants of uncertain significance
ROC	Receiver operating characteristic
PR	Precision–recall
DCA	Decision curve analysis
DDD	Deciphering Developmental Disorders
CNS	Central nervous system
SHH	Sonic hedgehog
FGF	Fibroblast growth factor
BMP	Bone morphogenetic protein
CHD	Congenital heart disease
NDD	Neurodevelopmental disorder
ABC	Aberrant Behavior Checklist
CARS	Childhood Autism Rating Scale
ADOS	Autism Diagnostic Observation Schedule
