# Supplementary material for: A Clinical Prediction Model for Genetic Risk in Children with GDD/ID: A Retrospective Study
Source: Pediatr Rep. 2025 Dec 19;18(1):1. doi: 10.3390/pediatric18010001 (PMC12821521; doi:10.3390/pediatric18010001)
Supplement: Supplementary file 1 [file pediatrrep-18-00001-s001.zip › pediatrrep-3964785-supplementary.pdf]

Supplementary Material

1. Supplementary Analyses on the Exclusion of ASD + GDD/ID Children

To further clarify the rationale and necessity of excluding children with ASD during model development, we conducted additional analyses to compare clinical characteristics and model performance between children with GDD/ID comorbid with ASD (ASD + GDD/ID) and those with isolated GDD/ID (GDD/ID-only). These supplementary results provide additional evidence supporting the appropriateness of excluding ASD cases in the primary modeling process.

1.1 Comparison of Clinical Characteristics Between ASD + GDD/ID and GDD/ID-only Groups

To compare the distribution of major clinical characteristics between children with ASD + GDD/ID and those with GDD/ID-only, χ^2 tests or Fisher's exact tests were performed. The two groups were compared in terms of gender, craniofacial malformations, skeletal abnormalities, skin and hair abnormalities, visceral abnormalities, epilepsy, physical development abnormalities, offspring of AMA, ART offspring, premature infant, and family history of ID. The results showed that children with ASD + GDD/ID had significantly lower frequencies of gender, craniofacial malformations, skin and hair abnormalities, visceral abnormalities, and physical development abnormalities compared with GDD/ID-only children, indicating distinct differences in clinical phenotypes between the two groups (Table S1).

Table S1. Univariate analysis comparing ASD + GDD/ID and ID-only groups.

Variable	GDD/ID-only (%)	ASD + GDD/ID (%)	*p*-value
Gender (male)	70.4	79.8	0.0012
Craniofacial malformations	24.5	7.4	<0.0001
Skeletal abnormalities	2.9	1.3	0.1112
Skin and hair abnormalities	5.6	0.6	0.0002
Visceral abnormalities	7.0	1.9	0.0008
Epilepsy	1.0	0	0.1225
Physical development abnormalities	11.0	4.2	0.0003
Offspring of AMA	0.6	1.0	0.6994
ART offspring	1.0	0.3	0.4665
Premature infant	1.6	1.0	0.5856
Family history of ID	3.8	4.2	0.7543

Note: ID = intellectual disability; ASD = autism spectrum disorder; GDD = global developmental delay; AMA = advanced maternal age; ART = Assisted Reproductive Technology.

1.2 Model Sensitivity Analysis Including Children with Co-occurring ASD

To assess the impact on the original model's predictive performance when children with ASD + GDD/ID were included, we conducted variable selection using LASSO regression followed by multivariable logistic regression (Figure S1, Table S2), and subsequently constructed a nomogram (Figure S2). The results showed that after including individuals with co-occurring ASD, the model's AUC decreased to 0.6745 (95% CI, 0.648-0.701)

(Figure S3), with a sensitivity of 0.484, specificity of 0.837, accuracy of 0.699 (Table S4), and a PR-AUC of 0.667 (Figure S4). Both model fit and decision curve performance declined, indicating that directly including ASD cases in the model substantially reduced predictive performance (Figure S5-S6, Table S5).

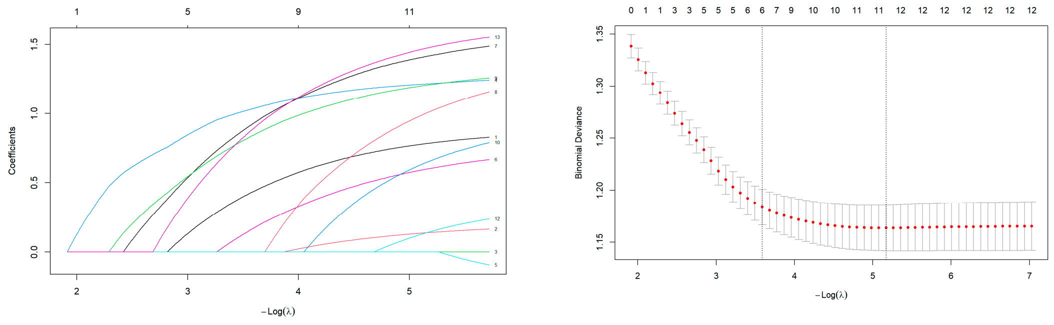

(**a**) (**b**)

Figure S1. LASSO Regression for Variable Selection. (**a**) Coefficient profiles of predictors. (**b**) Cross-validation for tuning parameter (λ).

Table S2. Multivariate Logistic Regression Analyses for Screening Predictors.

Variables	*β* (SE)	OR (95% CI)	*p*-value
Craniofacial abnormalities	1.227 (0.162)	3.41 (2.48–4.68)	<0.0001
Visceral abnormalities	1.529 (0.333)	4.62 (2.40–8.88)	<0.0001
Physical development abnormalities	1.244 (0.241)	3.47 (2.17–5.53)	<0.0001
Family history of ID	1.673 (0.353)	5.33 (2.67–10.66)	<0.0001

Note: ID = intellectual disability;

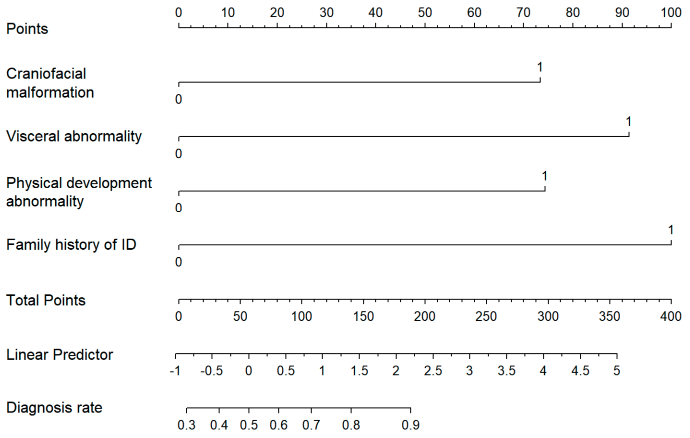

Figure S2. Nomogram for Genetic Risk in Children with GDD/ID.
Note: ID = intellectual disability;

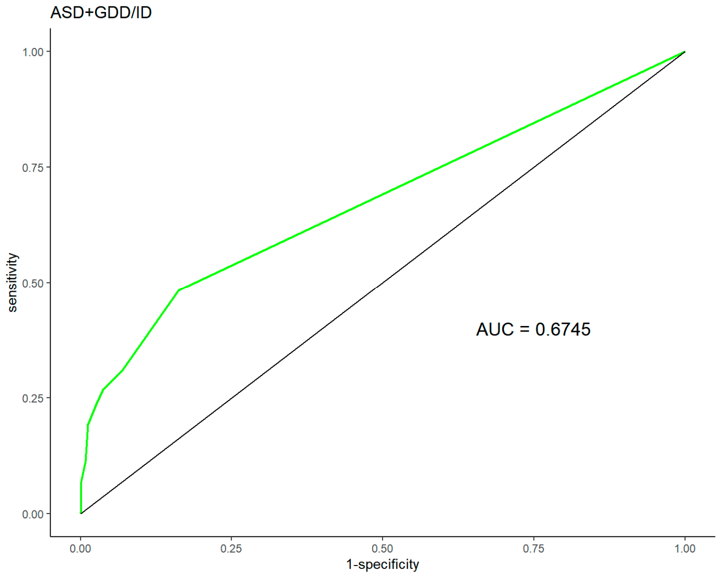

Figure S3. ROC Curves of the Model.
Note: AUC = area under the receiver operating characteristic curve.

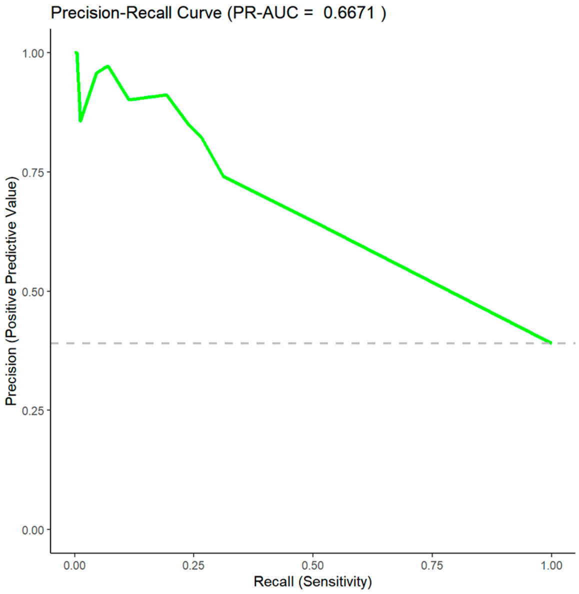

FigureS4. Precision-Recall Curves of the Model.

Table S3. Confusion Matrix of the Model.

	**Actual=0**	**Actual=1**
Predicted=0	633	250
Predicted=1	123	234

Note: 0 = genetic test negative; 1 = genetic test positive.

Table S4. Performance Metrics of the Model.

**Metric**	**Value**
AUC	0.6745

95% CI	0.648-0.701
Accuracy	0.699
Sensitivity	0.484
Specificity	0.837
PPV	0.656
NPV	0.717
PR-AUC	0.667
Threshold	0.423

Note: AUC = area under the receiver operating characteristic curve; CI = confidence interval; PPV = positive predictive value; NPV = negative predictive value; PR = precision–recall;

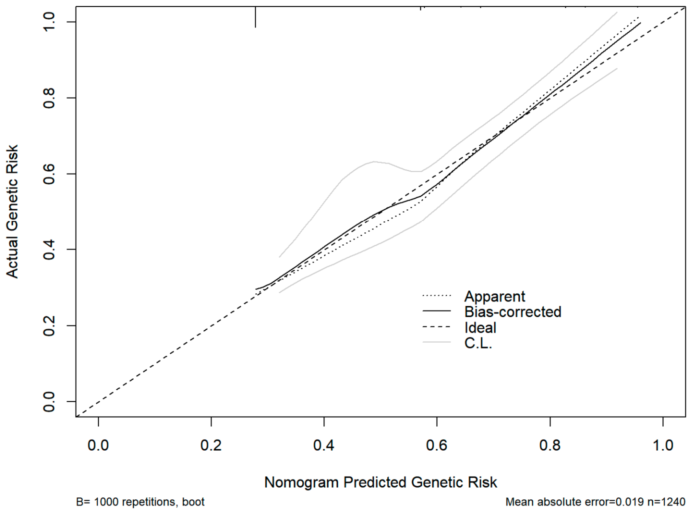

Figure S5. Calibration Curve for Predicting Probability.

Table S5. Calibration and Predictive Performance of the Model.

Metric	Value
Brier Score	0.202
Calibration Intercept	-1.78×10^{-14}
Calibration Slope	1.000
Calibration Slope 95% CI	0.841-1.168

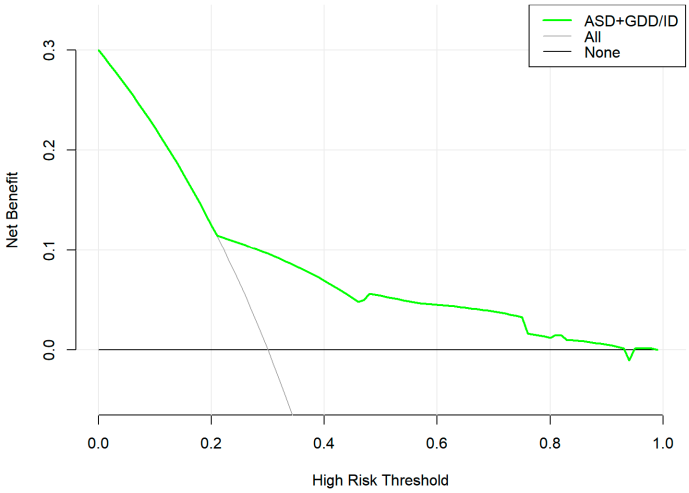

Figure S6. Decision Curve Analysis of the Model.

1.3 Summary and Interpretation

The supplementary analyses demonstrated that children with ASD + GDD/ID exhibit systematic differences in key clinical characteristics compared with GDD/ID-only children. Furthermore, including ASD cases directly in the predictive model substantially reduced its performance. These findings support the exclusion of ASD children during model development to ensure both the clinical relevance and statistical robustness of the model.
